# IgG Antibody 3D Structures and Dynamics

**DOI:** 10.3390/antib7020018

**Published:** 2018-04-19

**Authors:** Jacob White Jay, Brinkley Bray, Yaozhi Qi, Eseosaserea Igbinigie, Hao Wu, Jinping Li, Gang Ren

**Affiliations:** 1 Department of Biomedical Sciences, Mercer University School of Medicine, Savannah, GA 31404, USA; Jacob.White.Jay@live.mercer.edu (J.W.J.); Brinkley.Ann.Bray@live.mercer.edu (B.B.); qi_y@mercer.edu (Y.Q.); eseosaserea.grace.igbinigie@live.mercer.edu (E.I.); 2 Department of Clinical Laboratory, Lianyungang Maternal and Child Health Hospital, Lianyungang 222005, China; 3 College of Information Science and Technology, Beijing Normal University, Beijing 100875, China; 11132015314@bnu.edu.cn; 4 The Molecular Foundry, Lawrence Berkeley National Laboratory, Berkeley, CA 94720, USA

**Keywords:** single molecule 3D image, individual-particle 3D image, antibody structure, bispecific antibody, electron tomography, individual-particle electron tomography, IPET, 3D structure of IgG, antibody dynamics, antibody engineering, homodimer antibody, structure of bispecific IgG1

## Abstract

Antibodies are vital for human health because of their ability to function as nature’s drugs by protecting the body from infection. In recent decades, antibodies have been used as pharmaceutics for targeted therapy in patients with cancer, autoimmune diseases, and cardiovascular diseases. Capturing the dynamic structure of antibodies and characterizing antibody fluctuation is critical for gaining a deeper understanding of their structural characteristics and for improving drug development. Current techniques for studying three-dimensional (3D) structural heterogeneity and variability of proteins have limitations in ascertaining the dynamic structural behavior of antibodies and antibody-antigen complexes. Here, we review current techniques used to study antibody structures with a focus on the recently developed individual-particle electron tomography (IPET) technique. IPET, as a particle-by-particle methodology for 3D structural characterization, has shown advantages in studying structural variety and conformational changes of antibodies, providing direct imaging data for biomolecular engineering to improve development and clinical application of synthetic antibodies.

## 1. Introduction

Antibodies, or immunoglobulins (Ig), are glycoproteins that make up the humoral portion of the adaptive immune system and fight against pathogens such as viruses, bacteria, parasites, and diseased cells [1]. Antibodies share more than 90% of their identity in their primary sequences; they can be divided into five major classes including IgA, IgD, IgE, IgG, and IgM [2] based on their heavy chains, which differ in physicochemical and serologic properties as well as in their behavior as antigens themselves [3,4]. About 10–20% of proteins in the blood are IgG proteins. Due to their immense variability, plasma antibodies have more than 10 billion types, which can be classified into four subclasses: IgG1, IgG2, IgG3, and IgG4 [5], in the order of decreasing abundance in serum [2]. IgG1, for example, primarily responds to soluble and membrane protein antigens [6].

**Figure 1:**
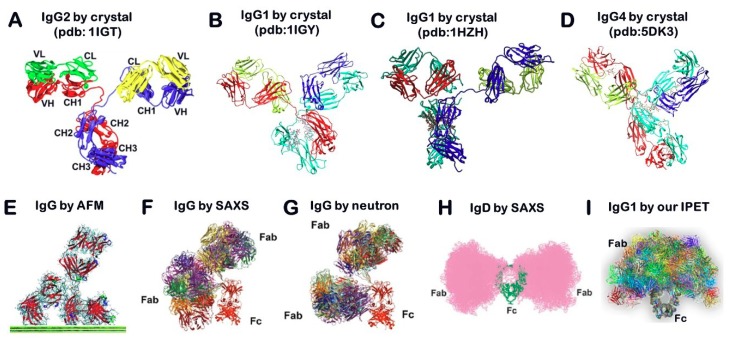
Structure and dynamics of antibodies. (**A**) The crystal structure of mouse IgG2a (PDB entry: 1IGT) shows that an antibody consists of two identical heavy protein chains (blue and red) combined with two identical light chains (green and yellow), which are composed of 7 (for constant domains) to 9 (for variable domains) β-strands (Copyright© Wikipedia). (**B**,**C**) However, the crystal structures of mouse IgG1 and human IgG1 (PDB entries: 1IGY and 1HZH) are different, especially for the Fab domains’ location and orientation. (**D**) The crystal structure of the full-length of the IgG4 antibody was also determined by the crystal structure and the hinge SS was deleted (PDB entry 5DK3). (**E**) Those structures are also different from the IgG revealed by atomic force microscopy (AFM) (Copyright© 2016 The Royal Society of Chemistry) [17], (**F**) small-angle X-ray scattering (SAXS), and (**G**) neutron scattering (Copyright© 2012 Elsevier Ltd.). This variation is also different from that of (**H**) IgD in solution revealed by SAXS (Copyright© 2005 Elsevier Ltd.) or (**I**) the structure and fluctuation of IgG1 obtained by negative-staining electron tomography (NS-ET) and individual-particle electron tomography (IPET) 3D reconstructions [16]. Copyright© 2015 the Authors, managed by Nature Publishing Group.

Antibodies contain two fragment antigen binding arms (Fab) that are formed from an N-terminal heavy chain variable domain (V_H_) and a light chain variable domain (V_L_) (Figure 1A). The V_H_ and V_L_ are linked together to form a monomer (H2L2 via inter- or intra-chain disulfide bonds (S-S)) [7,8]. Each heavy chain contains one V_H_ and three to four constant domains (CH1, CH2, and CH3 or Cγ1, Cγ2, and Cγ3). These V_H_ and V_L_ units lie together at the antigen-binding cleft, and the hinge region between CH1 and CH2 allows antibodies (IgGs) to display their flexibility upon antigen binding. The lower hinge region between CH2 and CH3 compose the fragment crystalline domain (Fc), which is responsible for IgG-Fc binding (FcγR, effector function), C1q (complement activation), and the neonatal Fc receptor (FcRn, homeostasis and placental transport, except for IgG2) [9]. Glycosylation of IgG1 mainly occurs on Asn-297 of the CH2 domains [10].

Monoclonal antibody production has shifted biological and pharmaceutical research as well as clinical therapeutics [11]. In 1975, Köhler and Milstein first used hybridoma technology to develop a mouse monoclonal antibody for combating kidney transplant rejection [12]. So far, more than 60 therapeutic monoclonal antibody products have been approved in the US or Europe, while nearly 100 antibodies are currently being tested in clinical trials [13,14]. First seen with the development of Humira (adalimumab) for treating rheumatoid arthritis, fully humanized antibodies have been generated by assembling lymphocyte V-region genes cloned to display Fab fragments on bacteriophage surfaces [15]. Today, fully humanized antibodies significantly improve the safety and efficacy of monoclonal antibody drug-based therapeutics and diagnostics, including antibody-conjugated drug delivery systems, oncoprotein-targeted cancer therapies, and immunotherapies for cardiovascular, neurodegenerative, and other diseases [16]. In the biopharmaceutical industry, ten of the fifteen top-selling drugs are protein biologics, and of these, five are monoclonal antibodies—the largest class of biotherapeutics—which represent at least 40% of the drugs in development today. A key limiting factor in the biopharmaceutical industry is the inability of current methods to characterize the structure of protein-based drugs fully and efficiently with sufficient precision and accuracy. Thus, we review several major imaging techniques for characterizing antibody structure and dynamics.

## 2. Methods for Characterizing Basic Antibody Structure

### 2.1. X-ray Crystallography

X-ray crystallography is a popular method for determining protein structure. In this approach, proteins are isolated, concentrated, and solidarized into crystal form. They are then exposed to an X-ray beam from which diffraction spots are processed as an electron density map to produce a three-dimensional (3D) structure [18]. Although hundreds of antibody structures are available through the Protein Data Bank (PDB), most of them are antibody fragments, typically the Fab arm with a binding pocket. Because of the natural flexibility of antibodies, the full-length structure of an antibody is difficult to determine using X-ray crystallography. To our knowledge, only four structures of IgG antibodies have been determined (PDB entries: 1IGT [19], 1IGY [20], and IHZH [21]). 1IGT is a mouse IGG2 with 3 hinge disulfide (SS) bonds, while human IGG2 has 4 SS bonds. 1HZH is human IgG1 with 2 SS bonds, same as 1IGY, a mouse IgG1. Although full-length IgG4 antibody was also determined by crystal structure, the hinge SS was more stabilized due to the conformational alteration of S228P mutation (PDB entry 5DK3) [14]. Four different conformations show a “Y”-shaped structure, however, the positional orientation and distribution of their Fab domains were different (Figure 1A–D). Considering all structures were determined from the solid state of the protein, their single rigid conformation may be insufficient to show crucial, solution-state dynamic structural features, such as fluctuations and torsions.

### 2.2. Atomic Force Microscopy (AFM)

AFM is a tool for imaging the surface topography of macromolecules through moving a sharp tip attached to a soft cantilever [22]. Although AFM allows for the imaging of antibodies and other biomolecules in their native environments, surface topography images insufficiently characterize the 3D structure with images potentially containing artifacts from molecular interaction with the supporting substrate. Using high-speed AFM, the innate fluctuation and dynamic nature of antibodies can be directly observed (Figure 1E) [17,23]. The main advantage of this technology includes the fact that it provides a tool for the quantitative determination of protein at nanometer resolution and the application of forces in the pico-Newton regime, which allows comparing the flexibility between proteins for the mechanical measurement of individual proteins. Moreover, improvements in methodology have allowed for video recording of the real-time dynamics of protein, such as antibodies “walking” on the substrate [23]. High-resolution AFM images of biomolecules and assemblies can reveal sub-molecular details with the ability to observe conformational changes directly, such as the binding of monoclonal antibodies to various partners [23].

### 2.3. Small Angle Scattering (SAS) Method

SAS, which includes small-angle X-ray scattering (SAXS) and small-angle neutron scattering (SANS), is a fast analogous method used to study the structural variety of objects in solutions at resolutions between 1 and 25 nm. Through the scattering curves of the biological sample exposed under X-ray or neutron beams, the experimental curves can be modeled for revealing the average particle size, shape, and surface-to-volume ratio. However, the fluctuation of IgGs revealed by X-ray scattering (Figure 1F) [24] and neutron scattering (Figure 1G) [24] showed a different distribution pattern from that of IgDs revealed by the SAXS technique (Figure 1H) [25] or AFM (Figure 1E) [17]. The study of antibodies in solution showed that the arms of IgGs are highly flexible and have a wide range of Fab–Fab and Fab–Fc angles, and the collective motions of the arms often contribute to the ability of IgGs to bind their antigens.

### 2.4. Nuclear Magnetic Resonance (NMR) Spectroscopy

NMR is an established and popular method for determining structural features for small molecules usually less than 40 kDa, [26] which is much smaller than the full-length of an antibody (usually 160 kDa). Using the ^15^N-, ^13^C-, and potentially ^2^H labeled protein for resonance assignment, this technique can be used to study antibodies in complexes in a weak and transient state to those in a very tight state. Monoclonal antibody formulations in glutamate solutions characterized by Kheddo et al. [27] explored the capability to characterize molecular translational diffusion and aggregation for ideal formulation [28]. However, the technical obstacles, such as attaining optimal solution concentration, close monitoring of the extent of self-association, and the high-level of expertise required for the process, explain why this methodology, which is sensitive to transient interactions between proteins, is viewed as a tool for providing useful complementary information for the formulation of monoclonal antibodies and other therapeutic proteins [29,30].

### 2.5. Transmission Electron Microscopy (TEM) Method for Imaging and 3D Reconstruction of Proteins

TEM can determine the structures of macromolecules and their complexes through 2D projections of objects. Several types of TEM techniques can be distinguished. Based on the sample preparation, the TEM technique can be called negative-staining (NS), positive-staining (PS), cryo-electron microscopy (cryo-EM), cryo-negative-staining (cryo-NS), and cryo-positive staining (cryo-PS). Based on 3D reconstruction methods, TEM approaches can be identified as electron crystallography, single-particle 3D reconstruction (a classification and averaging method), and electron tomography (ET). Recently, a study using the NS and ET technique [31] showed that the spatial distribution of Fab domains is much wider than previous reports (Figure 1I). In the following sections, we will briefly introduce some of these methods.

### 2.6. Negative-Staining (NS) and Positive-Staining (PS) Transmission Electron Microscopy (TEM) for Imaging Proteins

NS and PS are relatively older methods that were first developed by Errera, Welch, and Nuttal in the 1890s to enhance the certain surface features of microorganisms, examined with the aid of the light microscope [32]. In the 1950s, Farrant [33] and Hall [34] first used this method to image biological samples with TEM. By coating molecules with charged heavy metal salts, NS acquired high-contrast imaging of molecules via increased electron scattering by the heavy metal ions around the specimens [35] relative to the electron scattering by the less dense atoms of the proteins [36,37,38,39]. In the PS protocol, heavy metals bind to selective cell constituents to display an increased density of the electron beam on the sample. In PS, the TEM samples need to be washed in distilled water and air-dried to observe the positive density of the proteins [40,41].

### 2.7. Optimized Negative-Staining (OpNS) for Imaging Protein

**Figure 2:**
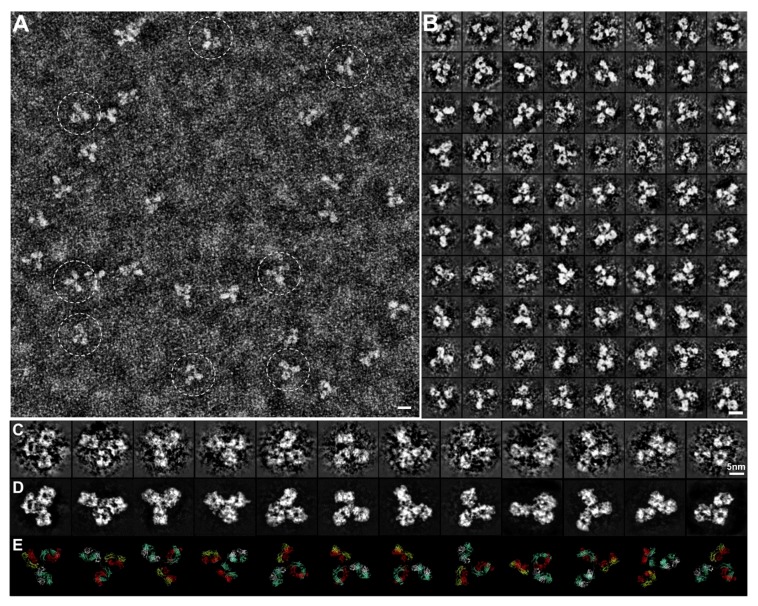
The Optimized Negative Staining (OpNS) Electron Microscopy (EM) images of IgG2 antibody particles. (**A**) Survey view of the IgG antibody particles imaged by OpNS EM. (**B**) Representative images of individual particles of antibody. (**C**) Zoomed-in views of selected individual particle images and (**D**) their corresponding denoised images are compared to (**E**) the crystal structure at a specific orientation. Copyright© 2015 the Authors, managed by Nature Publishing Group.

OpNS offers advantages over traditional NS, which typically provides relatively low-resolution images where stain-protein interactions may induce artifacts such as aggregation, flattening, and stacking [42,43,44]. For example in lipoproteins [35,45,46,47,48,49,50,51,52], a common artifact produced is rouleaux, where lipoprotein particles are stacked and packed together into a string [53,54,55,56,57,58]. To overcome these obstacles by reducing artifact formation and increasing resolution, an optimized negative-staining (OpNS) protocol has been developed [51,52,59,60,61,62,63,64,65,66,67,68,69]. In these studies, a specific type of staining reagent (such as Uranyl formate, UF) and relatively low salt concentration were identified as key parameters for avoiding rouleaux artifact formation, while the light-exposure prevention and small filtering (0.02 μm) methods were found as key parameters in obtaining high-resolution images of small and asymmetric proteins [31,52]. OpNS methodology has been used to examine several structure-known proteins such as cholesteryl ester transfer protein (CETP) [59], antibodies [31,60,61,62], GroEL and Protesome [63], and to determine the structure of unknown proteins, such as Contactin-associated Protein-like 2 (CNTNAP2) [64], Calsyntenin-3 [65], 84-base pair dsDNA conjugated with 5 nm nanogold [66], DNA origami [67], liposome-CETP complex [68], and lipoproteins and their forms when bound to an antibody [52,69,70,71]. The success in imaging these small proteins (40–200 kDa) provides evidence that the OpNS method is a useful tool to push the conventional EM boundary toward determining small and asymmetric protein structures, including antibodies (Figure 2).

### 2.8. Cryo-Electron Microscopy (cryo-EM) for Imaging Protein

Winning the Nobel Prize in Chemistry in 2017 [72], James Dubochet developed Cryo-EM which is an established and increasingly popular method used to analyze large molecules and molecule complexes (usually > ~100 kDa) at atomic resolution [73]. James Dubochet developed the cryo-EM method where buffer-based samples are rapidly frozen with a bath of liquid ethane cooled by liquid nitrogen. As a result, the molecules of water with supporting carbon films cannot form a crystalline lattice before being frozen into the amorphous form of an ice crystal, allowing the biomolecules in the sample to be retained in their native conformation [74]. A complementary advantage of cryo-EM, as compared to X-ray crystallography, is that cryo-EM allows for thousands of flash-frozen images of proteins that cannot be easily formed into large crystals. Recently developed high frame rate image detectors, such as the Gatan K2 direct detector camera, can provide a 10× higher frame rate than that of conventional cameras [75]. The high frame rate allows for tracking and correcting the drift of the cryo-EM images and increases the efficiency of signal-detecting to improve both the image contrast and resolution for large molecules [76].

### 2.9. Cryo-Negative-Staining (cryo-NS) and Cryo-Positive Staining (cryo-PS) for Imaging Protein

Cryo-NS and cryo-PS aid in enhancing the low contrast images of unstained biological particles imaged by traditional cryo-EM, especially for small proteins (<100 kDa). High defocus (typically several μm) is usually used to partially enhance the particle contrast, although this eliminates a certain percentage of structural information. Briefly, under higher defocus, the oscillation of the amplitude crosses zero more frequently and, as a result, the convolution between the contrast transfer function (CTF) and structure would cause an increased percentage of structural information to be permanently eliminated, limiting the imaging for small proteins. As a result, small proteins are challenging to study using this condition.

An alternative approach for imaging is largely needed to determine the structure of small proteins. Adrian et al. proposed an approach combining negative staining with cryo-EM, termed cryo-negative staining (cryo-NS), to overcome the low contrast under closer focus conditions [77]. In this method, the cryo-EM grid is washed quickly with a solution of 16% ammonium molybdate before rapidly freezing into a bath of liquid ethane. The cryo-NS images showed fine structural detail and the overall particle shape was consistent with the unstained cryo-EM image, supporting the advantages of the method.

**Figure 3:**
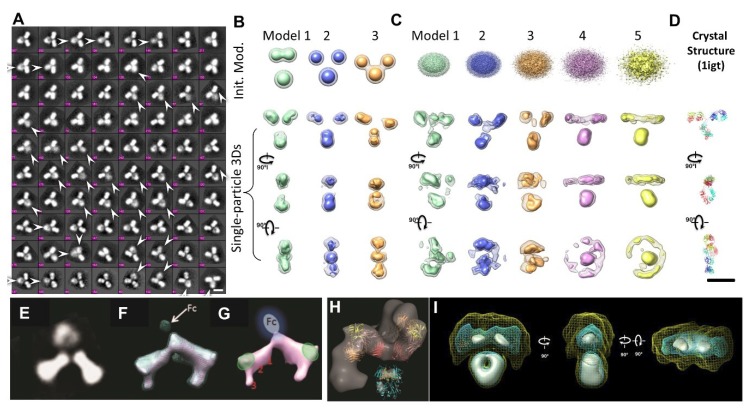
The artifacts shown in the averaged 2D images and 3D reconstructions of flexible antibody particles. (**A**) The fuzzy domains indicated by arrows in the selected reference-free 2D class averages of antibody images (a total of 11,373 particles). Bars: 100 Å. (**B**) The initial model bias and artifacts in the single particle 3D reconstructions of IgG antibody. The initial models of antibodies with three globular domains showed their bias in positions of the domains in the final 3D reconstruction. (**C**) While using Gaussian ellipsoids as bias-free initial models, the final 3D images showed incorrect structures by (**D**) comparing to the crystal structure. Copyright© 2015 the Authors managed by Nature Publishing Group. (**E**) A similar blurred 2D average also was apparent in the dual-variable-domain IgG molecules. (**F**–**H**) The single-particle 3D reconstruction (averaged particles) showed that the Fc domain was eliminated. Copyright© 2013 Landes Bioscience. (**I**) The averages of a hundred subvolumes of 3D reconstructions of antibody particles can also eliminate the domains. Copyright© 2015 the Authors managed by Nature Publishing Group.

As an alternative approach to cryo-NS, cryo-positive staining (cryo-PS) was reported by Zhang et al. for directly imaging the high-resolution structure of proteins imaged under near focus conditions [51,52]. For example, the high-resolution cryo-PS images of 53 kDa CETP contain remarkable secondary structural details of an individual molecule (Figure 3B) [6,59]. The cryo-PS method was developed by combining OpNS with conventional cryo-EM protocols. Instead of 16% ammonium molybdate, 1% Uranyl formate (UF) was used as a staining reagent. Since the cryo-PS image showed a reversed image contrast to that from the cryo-NS protocol and consistent contrast with that of the conventional cryo-EM image, it was named cryo-PS. The high-resolution cryo-PS images suggested that UF penetrates the molecular surface, which challenges the conventional understanding that staining can only occur on the outer surface of the protein. The mechanism of UF penetrating the molecular surface is unknown. One possibility is that the Uranyl cation binds to available protein carboxyl groups to enhance the electron scattering which is similar to the multiple isomorphous replacement (MIR) method in X-ray crystallography [78,79,80]. The cryo-PS protocol is a suitable backup method for cryo-ET and can be applied to study an individual macromolecule in solution, such as DNA origami [67].

### 2.10. Electron Crystallography for 3D Reconstruction of Protein

Electron crystallography is the first TEM technique that has been used to determine the atomic resolution structure of proteins, especially membrane proteins embedded in lipid bilayer [81,82]. This technique requires that proteins are oriented into 2D array. Through the merging of their electron diffractions and projection images acquired from different tilting angles, the atomic resolution structure of proteins can be determined. The limitation of this approach is similar to X-ray crystallography, in which adequate crystallization of proteins is required.

### 2.11. Single-Particle 3D Reconstruction for Averaging 3D Structure of Protein

Along with cryo-EM, single-particle 3D reconstruction for averaging the 3D structure of a protein was also a subject of the 2017 Nobel Prize in Chemistry and has been rapidly developed and used for protein structure determination at the atomic resolution over the past two decades. Although it was named “single-particle”, the 3D structure obtained is not from a single or an individual particle but averaged from thousands to tens of thousands of different particles. The advantage of the single-particle 3D reconstruction is to determine the molecular structure without prior requirement of crystallization. Therefore, the structure is more likely to be in its native state, instead of the solid state used for X-ray crystallography. Recent advances in direct detector technology for acquiring high-contrast images [75] and improvement of computer software algorithms [81,82] for precise classification and averaging now allow for the determination of the protein structures at atomic resolution [72]. The method has been also used for antibody 3D structure study [83,84]. The disadvantages of the method include (i) the sample needs to be relatively homogenous; (ii) particles were assumed to share one or few distinguishable identical conformations; (iii) 3D reconstruction depends on the initial model [85], such as that shown in (Figure 3B) [60]; (iv) the natural dynamics of protein can be mischaracterized [76]; (v) 2D projections/images are insensitive and insufficient to use to distinguish the conformation in 3D, such as the right-hand and left-hand objects [31]; (vi) no criteria can be used to confidently judge if the particles shared the same structure before averaging and classification; vii) potential artifacts can be produced in the 3D reconstruction of the flexible portion (Figure 3A,E) [30]; (viii) unexpectedly small dimensions are difficult to characterize (Figure 3F–H) [30]; (ix) certain domains can be eliminated (Figure 3H) [84]; or (x) resolutions can be of uneven distribution [83,84]. The absent domains/regions can even be shown in a near-atomic resolution single-particle 3D reconstruction, such as the ankyrin regions which disappeared in the atomic structure of TRPV1 [76].

### 2.12. Single-Particle Electron Tomography (ET) for Subvolume, Averaged 3D Structure of Proteins

Electron tomography (ET) is a method that has been used to study the structure of a single-instance large object, such as the section of a cell or bacteria, by producing an image from a series of viewing angles [86,87]. The 3D density map was reconstructed from these tilted images by a computer algorithm. The resolution of ET 3D reconstruction was believed to be too low to be used for the characterization of protein structure and dynamics [88]. To improve the 3D reconstruction resolution, a detour solution was proposed, in which hundreds to thousands of subvolumes that contained a single protein particle were aligned and averaged into a single 3D density map [89]. This approach showed its utility for imaging proteins that are structurally homogeneous with symmetry [88,90], including GroEL [91]. The subvolume averages can reduce the 3D noise and fill the missing wedge artifact to achieve near atomic resolution for the 3D reconstruction [92]. However, for proteins with substantial asymmetric and structural heterogeneity, the accuracy of the alignment and classification of those subvolumes can be disrupted by significant noise, missing wedge artifact, and the introduction of systematic reconstruction errors [89]. The errors in the classification and alignment can further influence the quality of the 3D averaging [31]. Moreover, for a continually changing structure, classifying particles into a limited number of groups is accomplished through human decision, which introduces subjectivity and bias that limits the objective understanding of the natural dynamics and equilibrium state of proteins in solution. For example, an averaged 3D map from a hundred subvolumes of 3D reconstructions of antibody particles would contain artifacts, such as shrinking the size of the Fab domains (Figure 3I) [60].

### 2.13. Individual-Particle Electron Tomography (IPET) for Single Molecule 3D Protein Structure

**Figure 4:**
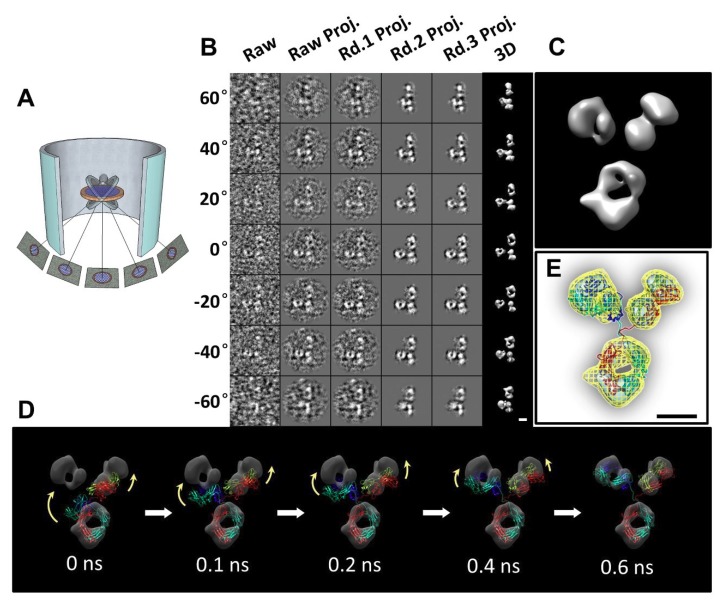
A 3D image of an individual particle of IgG1 antibody reconstructed by individual-particle electron tomography (IPET). (**A**) Schematics of the ET imaging of an individual particle of protein from a series of tilt angles. (**B**) The step-by-step process of the IPET 3D structure of an individual particle of IgG1. (**C**) The final IPET 3D reconstruction. (**D**) Docking the structural model by using molecular dynamics simulation. (**E**) The final IPET 3D reconstruction of a defined conformation of IgG1. Copyright© 2015 the Authors managed by Nature Publishing Group.

IPET is an experimental approach that can determine the 3D structure of an individual particle at nanometer resolution, opening the possibility to reveal the true, more accurate dynamic nature and fluctuations of molecules in solution (Figure 4) [6,8,18,34,60]. Comparing to conventional single-particle 3D reconstruction, IPET is the real single-particle 3D reconstruction that does not require a pre-established model, nor averaging from multiple molecules. In contrast, the traditional “single-particle” 3D reconstruction requires an initial model and/or averaging from hundreds to thousands of particles for obtaining a single averaged reconstruction. Moreover, IPET can tolerate the small tilt-errors or large-scale image distortions that often accompany the intermediate-resolution (1–5 nm) of single structural “snapshots” of molecules [31]. IPET improves the quality of the 3D reconstruction via several technical improvements by modifying the protocol for sample preparation (including OpNS [51,63]), methods for controlling data acquisition [93], the 3D reconstruction algorithm [31], and corrections for missing wedge. To avoid the influence of image distortion and tilt angle errors, the reconstruction algorithm (called focused electron tomography reconstruction (FETR)) reduces the reconstruction size for precisely aligning the tilt images under sets of dynamic filters and soft-boundary masks through local refinement iteration [31]. IPET has been used to study flexible proteins embedded in both vitrified ice (cryo-electron microscopy, cryo-EM) and heavy metal ions (negative-staining, NS). IPET 3D reconstructions from cryo-ET include 17 nm nascent HDL particles [31], 53 kDa CETP bound to liposome [68], DNA origami [67], and very low-density lipoproteins with their antibody binding complexes [69] in the resolution range from 3 to 10 nm. IPET 3D reconstructions were successfully obtained from high-contrast OpNS images, including 53 kDa CETP, 150 kDa IgG1 particles [31,60,61], peptide-antibody conjugates [61], an 84-base pair dsDNA bound to 5 nm nanogold particle [66], 88 kDa Calsyntenin-3 (C3) monomer [65], a 350 kDa C3 oligomer [65], 135 kDa Contactin-associated Protein-like 2 (CNTNAP2) [64], CNTNAP2 and 1.8 nm nanogold conjugates [64], CNTNAP2 and 5 nm nanogold conjugates [64], CNTNAP2-antibody complex [64], and a 60 kDa N-terminal domain of CNTNAP2 [64], in which the resolution can be improved up to 1.2 nm.

**Figure 5:**
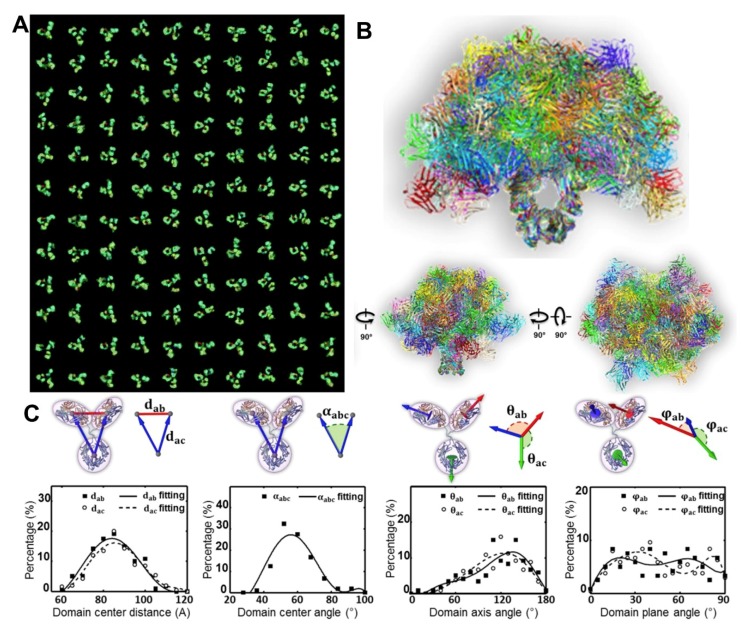
The IgG1 structural dynamics by individual-particle electron tomography (IPET). (**A**) 120 IPET 3D reconstructions from each individual particle of IgG1 and their docked structural models by using molecular dynamics simulation. (**B**) The overlap of 120 structural models revealed by 120 individual molecules, showing the difference in the structures. (**C**) Statistical analysis of the domain angle and distance distributions. Copyright© 2015 the Authors managed by Nature Publishing Group.

Although the IPET 3D has not been able to provide secondary structural features, it is still sufficient in allowing flexible docking of the crystal structure into IPET 3D by MD simulation under the chemical bond constraint and energy minimization conditions (Figure 4D,E). The structures revealed from this combined approach could answer some important biological questions, such as conformational changes of IgG1 domains induced by peptide conjugation [61]. IPET provides an exciting new opportunity for studying heterogeneous protein structures, dynamic characteristics (Figure 5), and equilibrium fluctuation by tracking chemical conformational changes and behaviors [71]. A review by Ericus et al. [94] has outlined the difficulties and solutions for EM regarding both hard and soft materials research for 3D reconstruction and visualization of sub-nanometer structures. In addition, it is reported that many methods can be utilized to overcome sample-based limitations in both physical and biological sciences with the motivation to innovate and advance large-scale solutions in a cooperative manner.

## 3. Applications of IPET 3D Reconstruction Approach on Antibody Studying

### 3.1. 3D Structure Variety of IgG1 Antibody by IPET 

**Figure 6:**
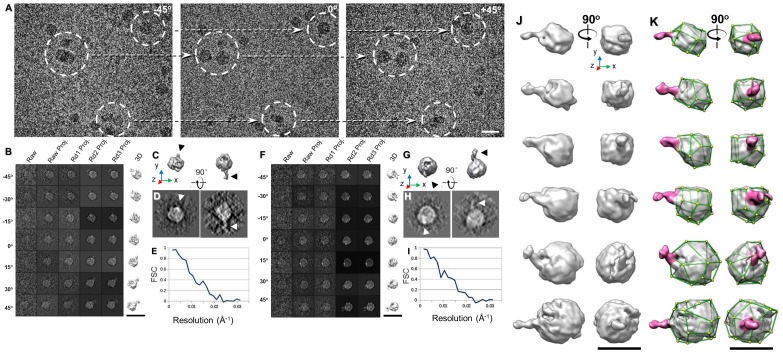
The IPET 3D structure of IgG1 bound to antigen in vitreous ice. (**A**) Cryo-ET images of IgG mixed with very-low-density lipoprotein (VLDL) particles. (**B**–**I**) IPET 3D reconstruction of two complexes of antibody bound to VLDL. The sample is embedded in vitreous ice. (**J**,**K**) Six presentative IPET 3D maps from six individual antibody-VLDL complexes (antibodies’ location were indicated in purple color). Copyright© 2016 by the American Society for Biochemistry and Molecular Biology, Inc.

Although other methods have provided invaluable structural data for antibodies and antibody-antigen complexes, IPET can visualize a single molecule in 3D at various conformations to provide more substantial evidence for the better characterization of antibody dynamics. Studies by Zhang et al. [60] better described the IgG1 flexibility and fluctuation using IPET reconstruction from OpNS ET images to obtain 3D density maps of a single protein at intermediate resolutions (1–3 nm) (Figure 4); thus, validating the applicability of the approach for understanding protein behavior and function. Such studies show that the combination of IPET and OpNS can expand the experimental approaches to the antibody structural dynamic nature of IgG1 (Figure 5) [60]. Other studies have highlighted the applicability of IPET 3D characterization of particle-particle interaction in vitreous ice, such as antibodies bound to human plasma very low-density lipoproteins (VLDL) (Figure 6) [69], and cholesteryl ester transfer protein (CETP) to liposome [68]. In the field of lipid research, cryo-EM has determined the polyhedral 3D structure of hydrogenous VLDL particles via IPET and has characterized the directional binding of cholesteryl ester transfer protein (CETP) to HDL and VLDL to improve the understanding of the role of VLDL in atherogenesis [69]. Such studies underscore the practicality of TEM alongside IPET to promote the understanding of dynamic antibody interaction [69].

The dynamics of IgGs depend on the hinge length, hinge SS patterns in between isotypes, and the conjugation and antigen binding for the same isotype. The numbers of disulfide bonds located between the hinge and the heavy chain regions are 2 for human IgG1 and IgG2, 4 for IgG4, and 11 for IgG3, although they each contain a total of 12 intra-chain disulfide bonds. Each disulfide bond is associated with an individual IgG domain; therefore, IgG3 has less flexibility for the formation of disulfide bond variants due to its higher number disulfide bonds. IgG4 also has non-classical disulfide bond structures. IgG1 has two proline residues and IgG4 contains one proline residue, which is unstable. The instability of the inter-heavy chain disulfide bonds of IgG4 allows it to be bispecific in vitro. The number of disulfide bonds and residues thus affects the flexibility of a hinge region and may affect the possible conformations of the Fab arms and Fc domains. The number of amino acids in hinge regions of IgGs is variable: 15 in IgG1, 12 in IgG2, 62 in IgG3, and 12 in IgG4. Although the hinge regions of both IgG2 and IgG4 contain 12 amino acids, the polyproline helix makes the IgG2 hinge even more rigid. In IgG3, the Fab fragments are relatively far from the Fc fragment, yielding greater flexibility. The flexibility of Fab arms relative to Fc domains varies between subclasses (IgG3 > IgG1 > IgG4 > IgG2) and this flexibility affects the antigen binding capacity and immune complex formation. It is also important to note that much of the limited literature on the functional characterization of structural dynamics and antibody stability of varying immunoglobulin isoforms comes from mutagenesis studies that have examined the effects of selective mutations of specific hinge-region residues through observation of the subsequent changes in antibody flexibility, stability, and binding, with disulfide patterns and hinge length being factors that potentially affect the dynamic nature of different human IgGs.

**Figure 7:**
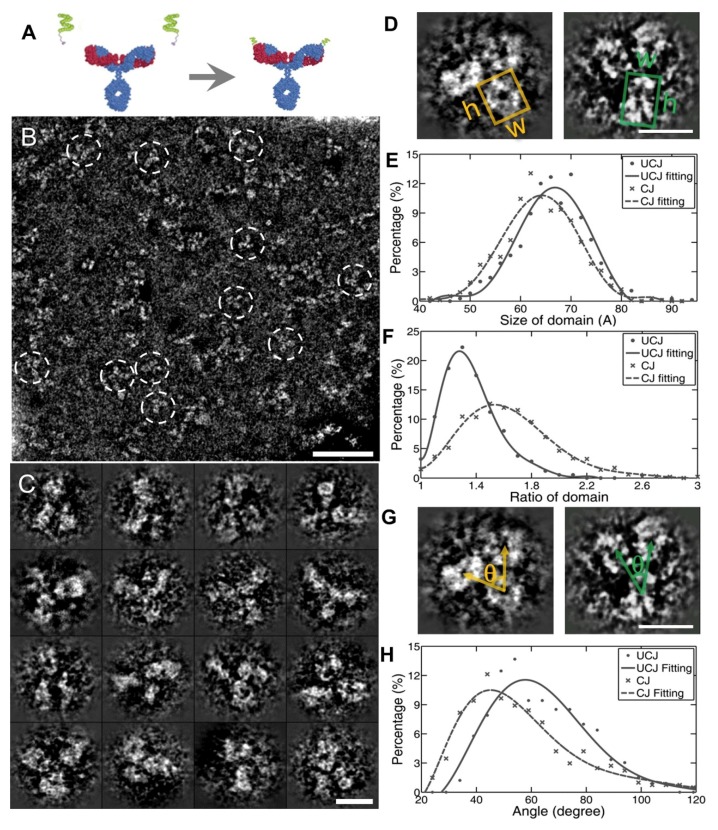
The peptide conjugated IgG1 structural dynamics and conformational changes in IPET. (**A**) The schematics of peptide conjugating with the IgG1 antibody. (**B**) The survey view of peptide conjugating IgG1. (**C**) The selected particles of IgG1 prepared by OpNS method. (**D**) Measuring the domain size and shape. The left is unconjugated, the right is conjugated. (**E**,**F**) Histograms of the domain size and shape. (**G**) Measuring the angle between two Fab domains, (**H**) the histogram of the angles. Copyright© 2013 the Authors managed by Nature Publishing Group.

Current understanding of antibody structural dynamics is rooted in several methods. Emphasized in this review, various TEM imaging techniques can be used to obtain the IgG dynamics. For example, directly measuring the angle of Fab-Fc-Fab from the acquired 2D images, as showed in the Figure 7H [61], mapping a 3D structure from the 2D image of each antibody for angle destitution analysis [83] and 3D reconstruction from each individual antibody for analysis is shown in Figure 5C [60]. Both the 2D and 3D analyses showed the same peak population of the antibody, while the 3D analysis shows a closer to Gaussian distribution and the 2D analysis showed a higher percentage of angles above the peak angle. Moreover, the role of antigen binding showed changes in the structure and dynamics of bound IGGs as showed in above VLDL-antibody complex [69] and below peptide antibody conjugates [29]. A detailed discussion was recently published by Chen et al. [83].

Characterization methods have been reported that use reduction, differential alkylation, liquid chromatography, and mass spectroscopy analyses to characterize the ranking order of disulfide bond susceptibility in recombinant antibodies. Modification of amino acids in the hinge region of IgG4 has been shown to produce structural alteration. Scapin et al. reported the therapeutic pembrolizumab structure, a human full-length IgG4 S228P anti-PD1 antibody at the 2.3-Å resolution [14]. They proposed that a shortened hinge region of pembrolizumab results in a new conformation by preventing the IgG4 arm exchange. Throughout the remainder of this review, we highlight the available imaging techniques that can be used to directly visualize antibody structures and better characterize dynamic conformational behaviors.

### 3.2. 3D Structure and Conformational Changes of Antibody Drug Conjugates (ADCs) by IPET

The rationale for using ADCs in pharmacology is explained by the ability to use the pharmacokinetics of an antibody to deliver an attached drug more efficiently [95]. The tagged antibody conjugates can be used for assessing drug effectiveness by quantifying their uptake into cells [96,97]. However, few papers highlight the nanometer-scale structural dynamics of ADCs due to the limitations of most imaging methods to clearly identify 3D structural changes after conjugation. The combined methods of IPET and OpNS, however, continue to provide evidence for visualizing and characterizing structural behaviors of ADCs.

A study by Tong et al. [61] used a modified peptide covalently linked to the complementary-determining region (CDR) loop on humanized h38C2 IgG1 (conjugated antibody: CovX-body by Pfizer CovX LLC) as a model to view images of conformational changes that occur because of conjugation (Figure 7). The OpNS protocols were used to obtain high-resolution images of the peptide-conjugated IgG1 compared to unconjugated IgG1 [71]. The domain size, shape, and fluctuations of conjugated- and unconjugated-IgG1 were quantitatively and statistically analyzed (Figure 7). Although the domain sizes remained constant, the images showed significant changes in the domain shape and fluctuations in the conjugated antibody. They found that the F_ab_^1^ − F_c_ − F_ab_^2^ angles became narrower in the conjugated IgG1, as compared to the unconjugated IgG1, indicating that the space between the F_ab_ regions likely became restricted because of peptide conjugation (Figure 7). This constriction may interfere with Fab binding to the antigen, which could also interfere with the Fc fragment binding to FCγII or FcRn.

**Figure 8:**
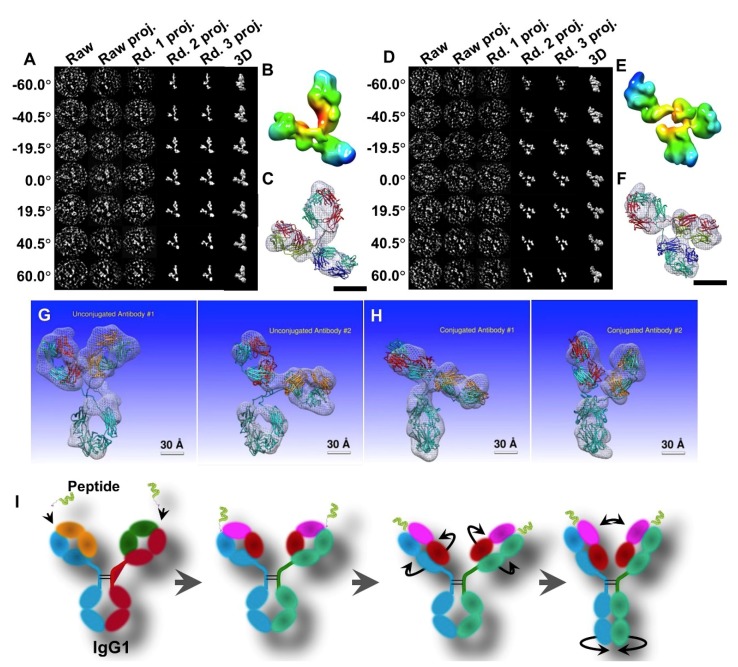
The IPET 3D maps of IgG1 and peptide conjugation. (**A**) The process of IPET 3D reconstruction of an individual particle of peptide conjugated antibody particle, (**B**) final IPET 3D density map, (**C**) and docked structural model using molecular dynamics simulation. (**D**) The process of another IPET 3D reconstruction, (**E**) its final IPET 3D density map, and (**F**) the docked model. (Copyright© 2015 the Authors, managed by Nature Publishing Group) (**G**) Two representative conformations of two unconjugated antibody particles. (**H**) Two representative conformations of two peptide conjugated antibody particles. (**I**) Schematics of the mechanism of the conformational changing induced by peptide conjugation.

The IPET approach was then used with the FETR algorithm to convert OpNS 2D images into reconstructed 3D models for each individual particle (Figure 8). The F_ab_^1^ − F_c_ − F_ab_^2^ domains of conjugated IgG1 showed an elongated, rod-like shape compared to unconjugated IgG1, leading to a more rigid and stiff structure. This indicates that the mechanism of peptide-induced conformational change is either due to the steric effects to compensate for both alpha helices of the peptide by reorienting the F_ab_ and F_c_ domains or due to the intertwining β-sheets of the F_ab_ region to trigger F_c_ domain alterations. This “trigger model” provides an explanation for the loss of the effector function in antibody-dependent cellular cytotoxicity (ADCC) (Figure 8) [95]. The conjugation-induced structural alterations likely caused changes in pharmacokinetics, making antibody clearance and removal physically challenging. Interestingly, the IPET protocol allows for a higher resolution image and more accurate angle measurements than the 2D images and further enhances the capability to monitor dynamic interactions of antibodies after drug delivery and facilitates more effective, efficient, and specific drug targeting (Figure 8). However, the current method is insufficient in providing any high-resolution structures and conformational changes regarding the complementarity-determining regions (CDRs) in response to antigen binding.

### 3.3. Bispecific Antibody Structure Variety by IPET

**Figure 9:**
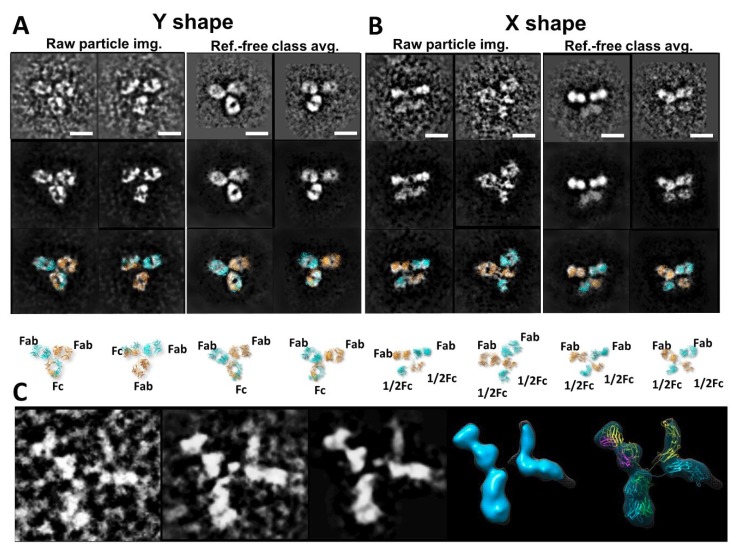
The IPET 2D and 3D images of IgG hole-hole homodimer (**A**) Two representatives of raw images of two Y-shaped antibodies and two representatives of reference-class averages of Y-shaped antibodies. (**B**) Two representatives of raw images of two X-shaped antibodies and two representatives of reference-class averages of X-shaped antibodies. Top to bottom, the rows show the original image, their denoised image, superimposed with the model, and the model. Copyright© 2017 American Chemical Society. (**C**) One example of 2D IPET 3D reconstruction of an individual-particle of X-shaped antibody. The order is raw image, projection of raw 3D, projection of final 3D, final IPET 3D, and docked model.

Because bispecific antibodies can simultaneously recognize two different targets, they are poised to revolutionize biotherapeutics through improved characterization and engineering. Unlike conventional monoclonal antibodies, knob-into-hole bispecific antibodies are subject to increased variant possibilities such as homodimerization in covalent and noncovalent forms. Recently, Zhang et al. [62] addressed a unique challenge of bispecific antibody production and characterization using the combination of EM and mass spectrum approaches to analyze storage- and pH-sensitive hydrophobic interaction chromatography (HIC) profile changes for a hole-to-hole homodimer bispecific antibody (Figure 9A,B). Compared to this unstable hole-hole homodimer Fc under different storage conditions, a bispecific heterodimer, characterized with the same methods and guided by knob-into-hole assembly, displayed stable conformation with homogeneous distribution, confirming its desired therapeutic quality while providing a better understanding of the homodimer and heterodimer variants. With advancements in the feasibility and accessibility of EM through methods such as OpNS and IPET 3Ds (Figure 9C), there is great potential to apply these high-throughput methods on a larger scale to accelerate research for the production of needed therapeutics.

## 4. Future

IPET has shown to be a powerful tool for the 3D imaging of a single protein, such as an antibody, and it offers promising evidence as a leading method for determining structural dynamics at a resolution near 1 nm. Although the other techniques and methods discussed here have shown their utility in characterizing antibody structure and antibody-antigen complex structure, distinct disadvantages remain that hinder their advancements. X-ray crystallography is limited by difficulties with sample crystallization, NMR spectroscopy presents difficulty visualizing large molecules, NS EM generates potential artifacts, cryo-EM presents difficulty imaging small proteins (<100 kDa) due to noise and potential 3D modeling bias, and AFM potentially produces surface artifacts from sample-substrate interactions. Despite the low resolution 3D images, IPET is the only known experimental approach for achieving a 3D image from a single small molecule, providing the capability to better characterize molecular conformational dynamics. These point to exciting possibilities for comprehensive antibody and antibody-antigen complex imaging for the advancement in applied molecular biology, immunology, and pharmacology. Importantly, IPET allows for the examination of samples at their near-native buffer state (such as, while embedded in vitreous ice) with an improved resolution without the need of coating with a heavy metal, often used in the NS approach. With better characterization of antibody dynamics and structural behavior, this technique, alongside other imaging methods, can be used to engineer more targeted approaches for the development of antibody therapeutics while improving efficiency and sustainability in addressing the continual, expanding need of treatment options for various diseases.

## Abbreviations

3D	three-dimensional
EM	electron microscopy
ET	electron tomography
IgG	Immunoglobulin-G molecule
IPET	individual-particle electron tomography
NS	negative-staining
OpNS	optimized negative-staining
cryo-EM	cryo-electron microscopy
